# Skeletal maturation evaluation: which is the reliability of dental calcification Demirjian method versus hand-wrist X-ray in growing subjects? A systematic review

**DOI:** 10.2340/aos.v83.40485

**Published:** 2024-05-03

**Authors:** Martina Ferrillo, Mario Migliario, Claudio Curci, Filippo Renò, Amerigo Giudice, Alessandro de Sire

**Affiliations:** a Department of Health Sciences, University of Catanzaro “Magna Graecia”, Catanzaro, Italy; b Department of Translational Medicine, University of Eastern Piedmont, Novara, Italy; c Department of Neurosciences, ASST Carlo Poma, Mantova, Italy; d Department of Health Sciences, University of Milan, Milan, Italy; e Department of Medical and Surgical Sciences, University of Catanzaro “Magna Graecia”, Catanzaro, Italy; f Research Center on Musculoskeletal Health, MusculoSkeletalHealth@UMG, University of Catanzaro “Magna Graecia”, Catanzaro, Italy

**Keywords:** Growth and development, dental maturity, skeletal maturity, Demirjian’s method, tooth calcification

## Abstract

**Objectives:**

This systematic review aimed at evaluating the reliability of dental maturation (DM) according to Demirjian method compared to hand and wrist maturation (HWM) to assess skeletal maturity (SM) in growing subjects, to identify the teeth and the corresponding mineralisation stages related to the pubertal growth spurt (PGS).

**Materials and Methods:**

PubMed, Scopus, and Web of Science were systematically searched until January 5^th^, 2024, to identify observational cross-sectional studies that assessed the reliability of Demirjian method compared to the HWM methods (i.e., Grave and Brown and Fishman) in growing subjects. The quality assessment was evaluated using the Joanna Briggs Institute (JBI) Critical Appraisal Checklist.

**Results:**

Out of 136 papers suitable for title/abstract screening, 19 included studies. Of them, 17 papers showed the reliability of Demirjian DM method compared to HWM Fishman and Grave and Brown methods to assess SM in growing subjects. According to JBI Critical Appraisal Checklist, 12 papers were high-quality studies and 7 papers were medium-quality studies.

**Conclusions:**

The mandibular second molar might be considered as the best indicator compared to other teeth and that the peak of growth occurs no earlier than stage F in females and stage G in males according to Demirjian method. Also, the mandibular canine might be analysed as indicator of SM in males, and results suggest that the peak of growth occurs no earlier than maturation stage F according to Demirjian method, only in male subjects. Further studies are needed to confirm these findings.

## Introduction

Skeletal discrepancies might have an impact on dentofacial features in growing patients, and the treatment timing plays a key role in achieving optimal results [1]. Indeed, the degree of skeletal maturity (SM) is a crucial aspect of orthodontic planning and the accelerated growth that occurs during the pubertal growth spurt (PGS) can significantly contribute to correct the dentoskeletal disharmonies [1–3].

Chronological age is not considered as an adequate indicator of skeletal age, because of variations in the timing of the PGS [4]. Thus, biologic age should be assessed evaluating the degree of maturation of different systems, and SM has most commonly been determined using hand-wrist radiographs evaluating the morphological changes and the ossification degree of hand and wrist bones [5–12]. However, to avoid additional X-ray exams, dental maturation (DM) assessment has been proposed to identify the PGS on panoramic radiographs, routinely used for diagnosis in orthodontics [7, 13, 14].

Dental maturation has been reported to be a potential predictor of SM and high correlation coefficients have been reported between dental calcification development and SM [13, 15–18]. However, it should be taken into account that high correlations with SM could represent a natural tendency because dental and skeletal structures are still considered as processes in progress. Moreover, these correlations could not provide information about which teeth and which mineralisation stage is satisfactory for the identification of the PGS.

In 2018, a meta-analysis by Bittencourt et al. [14] evaluated the correlations between DM and SM and concluded that studies suggested a strong correlation, but the authors pointed out a not clear association between teeth developmental stages and PGS, and also a high heterogeneity and methodology errors in the included studies. Other research has been conducted on this topic and a recent study concluded that the beginning of the PGS occurs when the roots of the canines or second premolars are almost totally mineralised [18], albeit other studies showed that the development of the second molar roots could be considered related to the PGS [13, 19].

However, to date, although the correlation between DM and SM in growing subjects has been proved, there is still a controversy in the scientific literature regarding the teeth and the corresponding stages of maturation that could identify the PGS.

Therefore, this systematic review aimed at evaluating the reliability of DM measurement according to Demirjian method compared to hand and wrist maturation (HWM) to assess SM in growing subjects to identify the teeth and the corresponding mineralisation stages related to the PGS.

## Methods

### Protocol and registration

This systematic review has been conducted according to Preferred Reporting Items for Systematic Reviews and Meta-analyses (PRISMA) guidelines [20], the protocol of this systematic review has been registered on the International Prospective Register of Systematic Reviews (PROSPERO) with number CRD42024499694.

### Search strategy

On January 5^th^, 2024, two authors systematically searched three different databases (PubMed, Scopus, and Web of Science) adopting the search strategy reported in Table 1. Furthermore, a manual search of the references of previous systematic reviews on similar topic was conducted as well.

**Table 1:** Search strategy for the systematic review.

***PubMed*** (“dental calcification” OR “dental age” OR “orthopantomography” OR “dental X-ray” OR “Demirjian” OR “Demirjian method”) AND (“hand-wrist” OR “hand-wrist X-ray” OR “hand-wrist radiographic” OR “hand-wrist analysis”) AND (“skeletal maturation” OR “maturation” OR “skeletal maturity” OR “maturity” OR “growth peak” OR “growth spurt” OR “pubertal growth peak” OR “pubertal growth spurt”) AND (“pubertal stage” OR “growing age” OR “pubertal” OR “growth” OR “children” OR “adolescents”)
***Scopus*** TITLE-ABS-KEY ((“dental calcification” OR “dental age” OR “orthopantomography” OR “dental X-ray” OR “Demirjian” OR “Demirjian method”) AND (“hand-wrist” OR “hand-wrist X-ray” OR “hand-wrist radiographic” OR “hand-wrist analysis”) AND (“skeletal maturation” OR “maturation” OR “skeletal maturity” OR “maturity” OR “growth peak” OR “growth spurt” OR “pubertal growth peak” OR “pubertal growth spurt”) AND (“pubertal stage” OR “growing age” OR “pubertal” OR “growth” OR “children” OR “adolescents”))
***Web of Science*** ((“dental calcification” OR “dental age” OR “orthopantomography” OR “dental X-ray” OR “Demirjian” OR “Demirjian method”) AND (“hand-wrist” OR “hand-wrist X-ray” OR “hand-wrist radiographic” OR “hand-wrist analysis”) AND (“skeletal maturation” OR “maturation” OR “skeletal maturity” OR “maturity” OR “growth peak” OR “growth spurt” OR “pubertal growth peak” OR “pubertal growth spurt”) AND (“pubertal stage” OR “growing age” OR “pubertal” OR “growth” OR “children” OR “adolescents”))

### Eligibility criteria

We evaluated for inclusion observational cross-sectional studies answering the question: ‘Which is the reliability of dental calcification Demirjian method versus hand-wrist X-ray in growing subjects?’.

Specifically, all studies were screened and considered as eligible according to the following PICO model:

P) Participants consisted of growing subjects;
I) Intervention consisted of Demirjian method for the SM assessment;
C) Comparator consisted of HWM methods commonly used in the clinical practice for the SM assessment (i.e., Grave and Brown and Fishman);
O) Outcome measure consisted of the reliability of Demirjian method compared to HWM for the SM assessment.

Exclusion criteria were: 1) studies with cleft lip/palate patients; 2) papers written in a language different from English; 3) other study designs (e.g., case reports, case series, and reviews); 4) full text unavailable (e.g., posters, conference abstracts, etc.); 5) book chapters; 6) animal studies.

### Study selection and data extraction

After removing the duplicates, two reviewers independently screened all the documents for title and abstract and then, for full text. A third author was asked to solve any disagreement by collegial discussion.

Then, two reviewers independently extracted data from eligible full-text papers utilising a customised data extraction form in Microsoft Excel. Key data were presented from each study relevant to the specific research questions. Any disagreement was solved by discussion between the two reviewers or by consulting a third reviewer.

The following data were extracted: 1) authors; 2) scientific journal; 3) publication year; 4) nationality of study participants; 5) population and number of patients; 6) age of subjects; 7) SM assessment methods; and 8) main findings.

### Quality assessment

To estimate the potential most relevant bias for the study, we used the Joanna Briggs Institute (JBI) Critical Appraisal Checklist for analytical cross-sectional studies [21]. Any disagreement was discussed until a consensus was reached with a third reviewer.

## Results

Out of 136 search results, 94 duplicates were removed, and 42 studies were considered as eligible for inclusion and screened for title and abstract. Out of these, we included 25 papers for full-text screening [22–40]. Six articles not respecting eligibility criteria were excluded by the systematic review.

**Figure 1:**
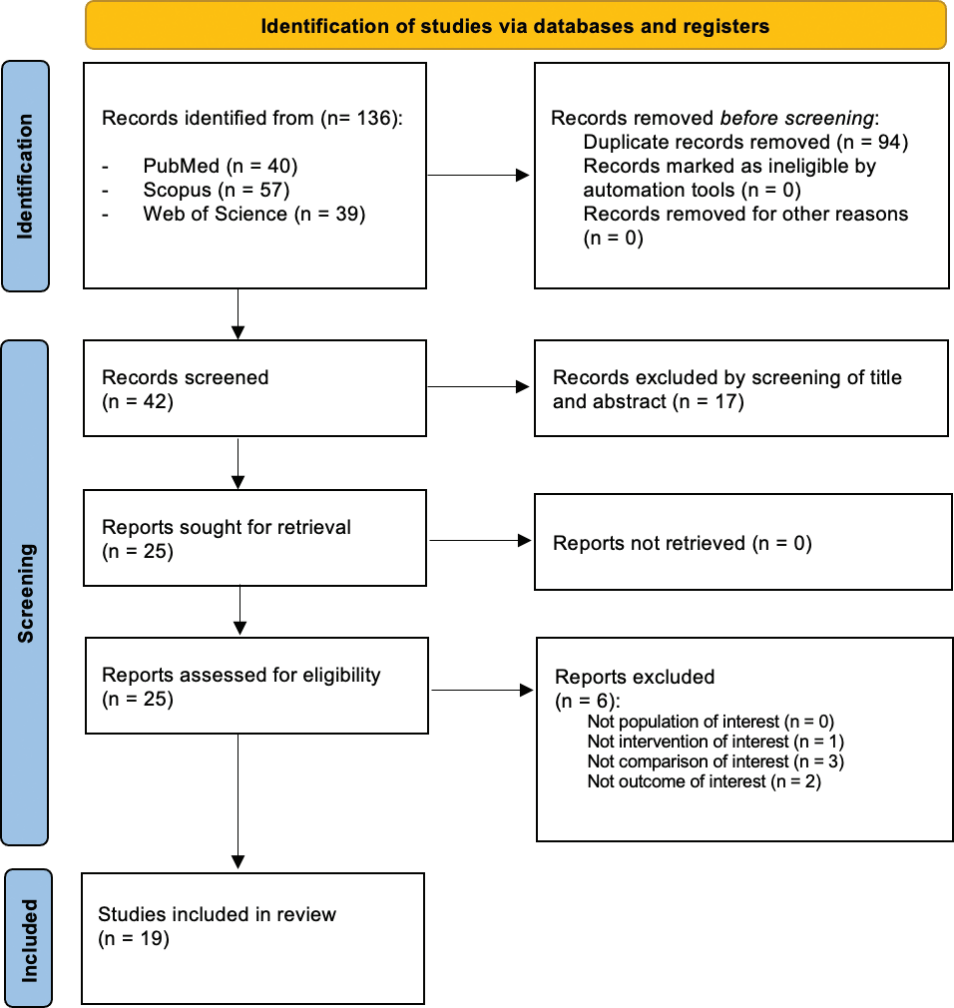
PRISMA 2020 Flow Diagram.

Thus, 19 papers were included in the synthesis, as depicted by PRISMA Flow Diagram in Figure 1. The main characteristics of the included papers are depicted in detail in Table 2.

**Table 2:** Main characteristics of the articles included in the present systematic review.

Authors	Journal	Publication year	Nationality	Population (M/F)	Age (years)	DM assessment method	HWM assessment method	Correlation between DM and SM
Camacho-Basallo et al.	Acta Odontol Scand	2017	Spain	202 (104 M / 98 F)	11–14	Demirjian	Grave and Brown	The authors examined the dental developmental stages of first and second premolars and first and second molars. No significant correlations were found between Demirjian method and HWM Grave and Brown method (*P* > 0.05).
Jourieh et al.	Biomed Res Int.	2021	Syrian	295 (145 M / 150 F)	9–14	Demirjian	Grave and Brown	The authors examined the dental developmental stages of mandibular left canine, first and second premolars, and second molar. The correlation between Demirjian method and HWM Grave and Brown were statistically significant for all teeth. The second molars showed the highest correlation both in males (*P* < 0:001) and females (*P* < 0:001).
Koçak et al.	J Orofac Orthop	2021	Turkey	297 (151 M / 146 F)	9–15	Demirjian	Grave and Brown	Hand–wrist and dental development stages of mandibular left canine, first and second premolars, and second molar showed a statistically significant relationship (*P* < 0.01) for both genders. The second molar reached the highest correlation coefficient with HWM.
Lopes et al.	Am J Orthod Dentofacial Orthop	2016	Brazil	491 (222 M / 269 F)	7–17	Demirjian	Grave and Brown	The authors examined the dental developmental stages of mandibular left canine, first and second premolars, and second molar. In females, only the second molar and the first premolar were statistically significant as predictors (*P* < 0.05). In males, only the first premolars were not a predictor of the maturation stage (*P* > 0.05).
Motghare et al.	J Forensic Dent Sci	2016	India	300 (150 M / 150 F)	7–20	Demirjian	Grave and Brown	The authors examined the dental developmental stages of mandibular canine, first and second premolars, and second molar. All the correlations between Demirjian and HWM were statistically highly significant (*P* < 0.0001). The second molars showed the highest relationship (0.865 in female; 0.760 in male).
Uysal et al.	Angle Orthod	2004	Turkey	500 (215 M / 285 F)	7–20	Demirjian	Grave and Brown	The authors evaluated the dental developmental stages of mandibular canine, first and second premolars, and second and third molars. All the correlations between Demirjian and HWM. The second molars showed the highest relationship (0.826 in female; 0.706 in male) and the canines showed the lowest correlation (0.691 in female; 0.633 in male).
Bagherpour et al.	J Clin Exp Dent	2014	Iran	52 (26 M / 26 F)	9–14	Demirjian	Fishman	The authors evaluated the correlation between the dental developmental stages of mandibular canines and second molars and the start of pubertal growth spurt (development of the sesamoid bones). They showed a significant correlation only for the left mandibular canine (*P* = 0.006) and the right mandibular canine (*P* = 0.014) only in males.
Camacho-Basallo et al.	Acta Odontol Scand	2017	Spain	202 (104 M / 98 F)	11–14	Demirjian	Fishman	The authors examined the dental developmental stages of first and second premolars and first and second molars. All the correlations between Demirjian method and HWM Fishman method were statistically significant (*P* < 0.001), with the exception of the first molars that showed a low significant correlation in female (*P* < 0.05) and not significant correlation in male (*P* > 0.05). The highest correlation was showed with second molars in female (0.711) and second molars in male (0.594).
Günen Yılmaz et al.	Acta Odontol Scand	2019	Turkey	717 (334 M / 383 F)	10–15	Demirjian	Fishman	The authors examined the dental developmental stages of mandibular canine, first and second premolars, and second molar. All the correlations between Demirjian and HWM were statistically significant (*P* < 0.01) and the pubertal growth spurt was best assessed through the dental evaluation of tooth 35.
Jeong et al.	Eur J Paediatr Dent	2022	Korea	447 (205 M / 242 F)	5–13	Demirjian	Fishman	The authors examined the dental developmental stages of mandibular canine, first and second premolars, and second molar. Significant correlations of dental calcification stages were found in all teeth examined (*P* < 0.05). The strongest correlations were found for the first premolars (0.780 among males; 0.812 among females).
Krailassiri et al.	Angle Orthod	2002	Thailand	361 (139 M / 222 F)	7–19	Demirjian	Fishman	The authors examined the dental developmental stages of mandibular canine, first and second premolars, and second and third molars. All the correlations between Demirjian method and HWM were statistically significant (*P* < 0.01). The CC indicates that the second premolar showed the highest correlation (0.69 in female; 0.66 in male) and the third molars showed the lowest correlation (0.31 in female; 0.47 in male).
Kumari et al.	Cureus	2022	India	120 (60 M / 60 F)	8–14	Demirjian	Fishman	The authors examined the dental developmental stages of mandibular canine, first and second premolars, and second molar. Results showed that correlations for canine, first premolar, second premolar, and molar were not significant.
Lecca-Morales et al.	Dental Press J Orthod	2017	Peru	78 (44 M / 34 F)	7–17	Demirjian	Fishman	The authors examined the dental developmental stages of mandibular canine, first and second premolars, and second molar. All the correlations between Demirjian method and HWM Fishman method were statistically significant (*P* < 0.001). The strongest correlation between DM stages and HWM stages was with second molars (0.792 in female; 0.800 in male).
Magat et al.	Imaging Sci Dent	2022	Turkey	284 (108 M / 176 F)	9–19	Demirjian	Fishman	The authors evaluated only the correlation between the dental developmental stages of third mandibular molars and the start of pubertal growth spurt and showed a moderate correlation (*P* < 0.05).
Mustafa et al.	Asian Biomed	2015	Saudi Arabia	60 (45 M / 15 F)	8–16	Demirjian	Fishman	The authors examined the dental developmental stages of mandibular canine, first and second premolars, and second molar. All correlations between Demirjian method and HWM Fishman method were statistically significant (*P* < 0.05) for male and not statistically significant (*P* > 0.05) for female. However, the Spearman’s CC were low also in male, ranging from 0.44 to 0.51 for the male.
Ojha et al.	J Forensic Dent Sci	2018	India	50 (25 M / 25 F)	8–14	Demirjian	Fishman	The authors examined the dental developmental stages of mandibular canine, first and second premolars, and second molar. All correlations between Demirjian method and HWM Fishman method were statistically significant (*P* < 0.05). CC ranged from 0.676 to 0.835 for males and from 0.812 to 0.882 for females. Authors concluded that canines reported the highest correlation in male (0.835) and the second molars in female (0.882).
Ojha et al.	Indian J Dent Res	2023	India	120 (60 M / 60F)	8–14	Demirjian	Fishman	The authors examined the dental developmental stages of mandibular canine, first and second premolars, and second molar. Results showed that correlations for canine, first premolar, second premolar, and molar were not significant.
Rebouças et al.	Dental Press J Orthod.	2021	Brazil	110 (60 M / 53 F)	9–15	Demirjian	Fishman	The authors examined the dental developmental stages of mandibular left canine and second molar. All the correlation coefficients were significant (*P* < 0.01) for the whole sample. Results showed that only the second molar was considered a significant predictor of skeletal development.
Sahin Sağlam et al.	J Orofac Orthop	2022	Turkey	422 (146 M / 276 F)	7- 14	Demirjian	Fishman	The authors examined the dental developmental stages of mandibular canine, first and second premolars, and second molar. The second molars showed the highest correlation in females (0.647) and males (0.550).
Yadav et al.	Natl J Maxillofac Surg	2017	India	120 (60 M / 60 F)	7–13	Demirjian	Fishman	The authors examined the dental developmental stages of mandibular canine, first and second premolars, and second molar. All the correlations between Demirjian method and HWM were statistically significant (*P* < 0.001). The second molars showed the highest correlation in female (0.845) and canines in male (0.755).

CC: Correlation coefficient; F: female; DM: dental maturity; HWM: hand–wrist maturation; M: male; SM: skeletal maturation.

### Correlation between Grave and Brown and Demirjian methods

Six papers [23, 26, 27, 31, 33, 39] compared Demirjian method with HWM Grave and Brown method. Only Camacho-Basallo et al. [23] showed no significant correlations (*P* > 0.05) between the two methods, whereas five studies [26, 27, 31, 33, 39] demonstrated significant correlations for all teeth (*P* < 0.01), and Lopes et al. [31] showed significant correlations for second molar and first premolar (*P* < 0.05) in females, and for all teeth except first premolars (*P* > 0.05) in males. More in detail, the second molar showed the strongest coefficient of correlation in these studies, with values ranging from 0.760 to 0.829 in male and from 0.790 to 0.880 in females.

### Correlation between Fishman and Demirjian methods

A total of 14 papers [22–25, 28–30, 32, 34–38, 40] compared Demirjian method with HWM Fishman method.

Of them, 12 studies [22–25, 28, 30, 32, 34, 35, 37, 38, 40] reported statistically significant correlations (*P* < 0.05) between Demirjian method and HWM Fishman method, and only two studies [29, 36] showed that correlations were not significant.

The studies showed differences in terms of highest correlation (expressed as CC) with teeth involved: Krailassiri et al. [28] reported second premolars in both sexes (0.69 in female; 0.66 in male); Lecca-Morales et al. [30] reported second molars in both sexes (0.792 in female; 0.800 in male); both Ojha et al. [35] and Yadav et al. [40] reported second molars in female (0.882 and 0.845, respectively) and canines in male (0.835 and 0.755, respectively); Jeong. et al. [25] reported strongest correlations for the first premolars (0.780 among boys; 0.812 among girls); Sahin Sağlam et al. [38] reported second molars in both female (0.647) and male (0.550).

Reboucas et al. [37] stated that highest correlations were reported for mandibular canine (0.712) and second molar (0.735). Bagherpour et al. [22] showed a significant correlation only for left (*P* = 0.006) and right mandibular canine (*P* = 0.014) only in males, although they evaluated only correlations for mandibular canines and second molars. Camacho-Basallo et al. [23] reported significant correlations (*P* < 0.001) between the two methods with the exception of the first molars that showed a low significant correlation in female (CC = 0.263; *P* < 0.05) and not significant correlation in male (CC = 0.187; *P* > 0.05). Magat et al. [32] evaluated only third molars and found only a moderate correlation between the dental developmental stages of third mandibular molars and the start of PGS (*P* < 0.05) in both males and females.

### Evidence of quality and risk of bias

In the present systematic review, the risk of bias of the included studies was analysed using the JBI Critical Appraisal Checklist for analytical cross-sectional studies [21].

Twelve papers were high-quality studies and seven papers medium-quality studies. Further details about the quality assessment of each study included in this systematic review are shown in Table 3.

**Table 3:** Quality assessment of the studies included in the present systematic review.

Articles	Criteria for the quality scoring								Score	Quality level
	Q1	Q2	Q3	Q4	Q5	Q6	Q7	Q8		
Bagherpour et al. (2014)	N	Y	Y	Y	Y	Y	Y	N	6	Medium
Camacho-Basallo et al. (2017)	Y	Y	Y	Y	Y	Y	Y	N	7	High
Günen Yılmaz et al. (2019)	Y	Y	Y	Y	Y	Y	Y	Y	8	High
Jeong et al. (2022)	N	Y	Y	Y	Y	Y	Y	Y	7	High
Jourieh et al. (2021)	Y	Y	Y	Y	Y	Y	Y	N	7	High
Koçak et al. (2021)	Y	Y	Y	Y	Y	Y	Y	N	7	High
Krailassiri et al. (2002)	Y	Y	Y	Y	Y	Y	Y	N	7	High
Kumari et al. (2022)	N	N	N	Y	Y	Y	Y	N	4	Medium
Lecca-Morales et al. (2017)	Y	Y	Y	Y	Y	Y	Y	N	7	High
Lopes et al. (2016)	Y	Y	N	Y	Y	Y	Y	Y	7	High
Magat et al. (2022)	N	N	Y	Y	Y	Y	Y	N	5	Medium
Motghare et al. (2016)	N	N	N	Y	Y	Y	Y	N	4	Medium
Mustafa et al. (2015)	Y	Y	Y	Y	Y	Y	Y	N	7	High
Ojha et al. (2018)	N	N	N	Y	Y	Y	Y	Y	5	Medium
Ojha et al. (2023)	Y	Y	N	Y	Y	Y	Y	N	6	Medium
Rebouças et al. (2021)	Y	Y	N	Y	Y	Y	Y	Y	7	High
Sahin Sağlam et al. (2002)	N	N	Y	Y	Y	Y	Y	Y	6	Medium
Uysal et al. (2004)	Y	Y	Y	Y	Y	Y	Y	N	7	High
Yadav et al. (2017)	Y	N	N	Y	Y	Y	Y	N	4	Medium

Joanna Briggs Institute Critical Appraisal Checklist for analytical cross-sectional studies. Legend: Q1. Were the criteria for inclusion in the sample clearly defined?; Q2. Were the study subjects and the setting described in detail?; Q3. Was the exposure measured in a valid and reliable way?; Q4. Were objective, standard criteria used for measurement of the condition?; Q5. Were confounding factors identified?; Q6. Were strategies to deal with confounding factors stated?; Q7. Were the outcomes measured in a valid and reliable way?; Q8. Was appropriate statistical analysis used?

N: no; Y: yes; U: unclear; N/A: not applicable.

## Discussion

The main finding of this systematic review was that dental calcification according to Demirjian method might be considered reliable to assess SM in growing subjects.

Specifically, among the 19 studies included in the present systematic review, 12 studies [22–25, 28, 30, 32, 34, 35, 37, 38, 40] reported statistically significant correlations between Demirjian method and HWM Fishman method and 5 studies [26, 27, 31, 33, 39] demonstrated significant correlations between Demirjian method and HWM Grave and Brown method. Only 2 studies [29, 36] showed that correlations were not significant.

### Mandibular canine and skeletal maturation

In terms of correlation between the dental developmental stages of mandibular canines and skeletal maturation, the results could suggest that the maturation of the mandibular canine may be related to the onset of the pubertal growth peak in male subjects.

Indeed, Lopes et al. and Bagherpour et al. [22] showed that the mandibular canine was not statistically significant as predictors of the maturation stages for girls (*P* > 0.05), whereas they showed a significant correlation between the start of PGS and the developmental stages of the mandibular canine in male subjects (*P* < 0.05). Accordingly, Ojha et al. [35] and Yadav et al. [40] showed that all correlations between Demirjian method and HWM Fishman method were statistically significant (*P* < 0.05), with a CC ranging from 0.676 to 0.845. Moreover, Ojha et al. [35] concluded that canines reported the highest correlation in male subjects (CC = 0.835) and Yadav et al. [40] concluded the same, reporting that the canines showed the highest correlation in in males (CC = 0.755).

Uysal et al. [39] found that 80% of male subjects in MP3cap stage according to Grave and Brown methos presented the mandibular canine in calcification stage H, and the 20% in stage G. No canines in stages D, E, and F were found in MP3cap stage.

Using the same HWM SM method, Motghare et al. [33] reported that 100% of male subjects had mandibular canine in stage H and no canines in stages D, E, F, and G were found in MP3cap stage.

Accordingly, Krailassiri et al. [28] found that 77.8% of subjects in MP3cap stage according to Fishman presented the mandibular canine in calcification stage H, and the 14.8% in stage G. No canines in stages D and E were found in MP3cap stage.

Rebouças et al. [37] reported that during the onset of the growth spurt, 80.9% of the subjects had the canine in stage F and G and during the peak, 92.9% had the canines in stage G and H.

The same findings were previously reported by Chertkow [41], Chertkow and Fatti [42] and Coutinho et al. [43]. The authors showed a high correlation between mandibular canine calcification and SM indicators, and stated that the skeletal maturation phase can be determined by the mandibular canine mineralisation phase.

Thus, the results of this systematic review suggest that peak growth occurs no earlier than the mandibular canine maturation stage F in males.

### Mandibular second molar and skeletal maturation

Analysing the correlation between mandibular second molar developmental stages and skeletal maturation, Lopes et al. [31] showed that the second molar was statistically significant as predictors of the maturation stages (*P* < 0.05). Specifically, the second molar mineralisation stage was reported to have 4.34 times (in females) and 6.8 times (in males) more chance to predict the skeletal maturation than the other teeth. Also, Koçak et al. [27], Lecca-Morales et al. [30], Mothgare et al. [33], Uysal et al. [39], and Yadav et al. [40] showed that the second molar reached the highest correlation coefficient with HWM evaluated using Grave and Brown method (0.826 to 0.877 in female; 0.706 to 0.790 in male).

Ojha et al. [35] showed that all correlations between Demirjian method and HWM Fishman method were statistically significant (*P* < 0.05), with a CC ranging from 0.676 to 0.835 for males and from 0.812 to 0.882 for females. Furthermore, the authors concluded that second molars reported the highest correlation in female (CC = 0.882).

Analysing the DM stages, Lopes et al. [31] demonstrated that the second molars mineralisation stages F and G appeared mostly in females and the mineralisation stage G in males at the peak. Accordingly, Rebouças et al. [37] reported that the majority of second molars were identified in stages F or G in females and stages G or H among the boys.

Furthermore, Koçak et al. [27] showed that the second molar stage G in the boys and stages G–H in the girls showed the highest percentage distribution at the peak. Camacho-Basallo et al. [23] showed that Fishman’s method was significantly associated with all stages of maturation in boys and girls, and reported that mandibular second molar stages F-G appeared in 66.3% of both males and females.

Thus, the results of this systematic review suggest that peak growth occurs no earlier than the mandibular second molar maturation stage F in females and stage G in males.

These results are in line with an interesting study by Oyonarte et al. [13] The authors conducted a longitudinal study analysing one hand and wrist radiograph and one 45° oblique radiograph from each side, in 60 subjects at age 6, 9, 12, 14, 16, 18, and 20 years. Moreover, they collected annual records of height and weight, and identified the onset of the pubertal growth peak. The authors showed high levels of correlation between Fishman and Demirjian methods and pubertal growth peak. Specifically, the stage F for females and stage G for males were identified as cutoff points between pre-pubertal and post-pubertal ages.

### Mandibular premolars and skeletal maturation

In terms of correlation between the dental developmental stages of mandibular premolars and skeletal maturation, Jeong et al. [25] found that the strongest correlations were found for the first premolars (*r* = 0.780 among males; 0.812 among females). On the other hand, Jourieh et al. [26] reported that the first premolars showed the lowest correlation (*P* < 0.001) in both genders.

Günen Yılmaz et al. [24] stated that the PGS was best assessed through the dental evaluation of second premolar, and accordingly, Krailassiri et al. [28] indicated that the second premolar showed the highest correlation (0.69 in female; 0.66 in male).

### Mandibular third molar and skeletal maturation

Analysing the correlation between third mandibular molar developmental stages and skeletal maturation, Magat et al. [32] found a moderate correlation (*P* < 0.05) in both males and females. Krailassiri et al. [28] showed that all the correlations between Demirjian method and HWM were statistically significant (*P* < 0.01), but the third molars showed the lowest correlation (0.31 in female; 0.47 in male). Similarly, Bagherpour et al. [22] and Uysal et al. [39] showed a significant correlation between the start of PGS and the developmental stages mandibular third molar, but this tooth showed the lowest correlation compared to the other teeth.

Thus, the results showed a poor association between the third molar calcification and the SM, as previously reported [41, 44]. This great variation in tooth development may adversely affect the identification of the relationship between dental and skeletal development.

This systematic review had some limitations that should be taken into consideration. The inclusion of only cross-sectional studies hinders the assessment of growth on longitudinal analysis. Moreover, the heterogeneity of the teeth assessed in the different included studies could have affected the results.

## Conclusions

Taken together, the findings of this systematic review showed the reliability of Demirjian dental calcification method compared to HWM Fishman and Grave and Brown in growing subjects to assess SM.

Moreover, the data analysis of the included studies could suggest that the mandibular second molar might be considered as the best indicator compared to other teeth and that the peak of growth occurs no earlier than stage F in females and stage G in males. Also, the mandibular canine might be analysed as indicator of SM in males, and the results suggest that the peak of growth occurs no earlier than maturation stage F in male subjects.

Further studies are needed to confirm these findings in order to improve the knowledge on the correlation between dental and skeletal development also using different methods that could be useful of dentists and physicians involved in the management of growing subjects.
